# The spillover effect of work connectivity behaviors on employees' family: Based on the perspective of work-home resource model

**DOI:** 10.3389/fpsyg.2023.1067645

**Published:** 2023-02-09

**Authors:** Hui He, Dan Li, Yuanyuan Zhou, Puliang Zhang

**Affiliations:** ^1^School of Business Administration, Zhongnan University of Economics and Law, Wuhan, China; ^2^School of Business, Xinyang Normal University, Xinyang, China

**Keywords:** proactive work connectivity behaviors, passive work connectivity behaviors, self-efficacy, ego depletion, family support, family harmony

## Abstract

With the rapid development of mobile communication technology, work connectivity behaviors are becoming increasingly pervasive, which has gradually attracted extensive attention from scholars and practitioners. According to the work-home resource model, we propose a theoretical model that proactive/passive work connectivity behaviors induce family harmony through self-efficacy and ego depletion, and we explore the moderating role of family support in this relationship. Based on survey data collected from 364 questionnaires using a three-wave time-lagged design, the results show that: (1) Proactive work connection behaviors have a negative effect on family harmony; Passive work connection behaviors have a negative effect on family harmony. (2) Self-efficacy plays a suppressing role in the relationship between proactive work connection behaviors and family harmony. (3) Ego depletion plays a mediating role in the relationship between passive work connectivity behaviors and family harmony; (4) Family support not only positively moderates the relationship between proactive work connectivity behaviors and self-efficacy, but also moderates the suppressing effect of proactive work connectivity behaviors on family harmony through self-efficacy; (5) Family support not only negatively moderates the relationship between passive work connectivity behaviors and ego depletion, but also moderates the mediating effect of passive work connectivity behaviors on family harmony through ego depletion. The above results can broaden our understanding of the effect of work connectivity behaviors and provide some inspiration for how to optimize the management strategy of employees' work connectivity behaviors.

## Introduction

With the development and popularity of communication technologies such as smartphones, mobile office computers, and wireless networks, it is becoming more pervasive for employees to use electronic communication tools to deal with work during non-working hours (Reinke and Ohly, 2021). In this context, work connectivity behaviors emerged, which is a new type of interaction between the individual, team, and organizational work (Khalid et al., 2021). The generation of work connectivity behavior relies on information technology innovation, which refers to the behavior of individuals to handle work or participate in workplace social interaction through communication devices and technologies during non-working hours (Richardson and Benbunan-Fich, 2011; Huo et al., 2022). Since the concept was proposed, scholars have successively explored the impact of work connectivity behaviors on employees' family outcomes. Throughout the current research situation, scholars have proposed two schools of view. Research on positive effects shows that the attribute of work connectivity behaviors is similar to job resources, enabling individuals to simultaneously address the dual needs of family and work, which is conducive to promoting work-family enrichment (Derks et al., 2016; Ma et al., 2016; Carvalho et al., 2021). Compared with the limited positive effects, more studies show that work connectivity behaviors generate a negative impact on families of individuals. Studies on negative effects show that the attribute of work connectivity behaviors is similar to job demands, which objectively increase employees' working hours and work intensity, and will occupy individuals' time and energy that should have been used for family life, thus leading to work-family conflict (Richardson and Thompson, 2012; Ragsdale and Hoover, 2016; Khalid et al., 2021).

Most of the previous studies have explored the effect and mechanism of work connectivity behaviors from a single perspective (i.e., positive or negative), and the outcome variables of its effect focused predominantly on work-family enrichment or work-family conflict, rather than specific outcome variables in the family domain. Therefore, the influence of work connectivity behaviors on specific outcomes in the family domain needs to be further explored. Family harmony is considered to be the sweet existence in family relations, emphasizing the closeness, harmony, cooperation, and interdependence among family members (Kavikondala et al., 2016), which is crucial to individuals' mental health and wellbeing (Ip, 2014). Therefore, in the context of “round-the-clock availability” (Ren et al., 2021), it is worth discussing how work connectivity behaviors affect family harmony. Moreover, on the basis of the paradox perspective, previous studies conducted a priori classification of work connectivity behaviors defined as job resources or job demands, and investigated it's positive or negative effects (Richardson and Thompson, 2012; Ter Hoeven et al., 2016). This classification oversimplifies the attribute of work connectivity behaviors and ignores the subjective motivation of employees. Relevant studies have pointed out that the way employees treat work will affect the impact of work on individuals (Schulte-Braucks et al., 2019). Therefore, as a typical boundary crossing behavior between work and non-work, how work connectivity behaviors will affect employees and their families depends on how employees treat such behavior (Huo et al., 2022). Based on previous studies, we consider the willingness of employees to participate in work connectivity behaviors and further divide work connectivity behaviors into proactive connectivity behaviors and passive connectivity behaviors from the perspective of subjective motivation (Huo et al., 2022). Furthermore, the impact of different types of work connectivity behaviors on employees' family outcomes have been insufficiently explored.

To solve the above questions, based on the Work-home Resources Model (W-HR), we explore the mechanism and boundary condition of proactive work connectivity behaviors and passive work connectivity behaviors on employees' family harmony. The W-HR model stresses personal resources as a link to connect the demands and resources in one domain with the outcomes in another domain, and systematically explains the causal logic and boundary conditions behind the work-family relationship (ten Brummelhuis and Bakker, 2012). The model indicates that in resource-based experiences, personal resources will gain and produce positive family outcomes. Conversely, in a demanding experience, personal resources will be depleted, resulting in negative family outcomes. According to the theoretical relevance and the suggestions of previous studies (Richardson and Benbunan-Fich, 2011; Schaufeli and Taris, 2014; Kang and Peng, 2019), we choose self-efficacy to represent the resource gain mechanism, and ego depletion to represent the resource loss mechanism, to fully reveal the “black box” of the impact of work connectivity behaviors on family harmony. In addition, the W-HR model also points out that under different situational resource conditions, there are differences in the degree of resources gained or depleted by employee behaviors, which in turn have different effects on family relationships (ten Brummelhuis and Bakker, 2012). Yet, the current work-family research has mostly focused on work support, such as leadership support and organizational support (Hammer et al., 2016; Kang and Peng, 2019), largely overlooking the role of family support in the realization of positive work-family relationships. Therefore, we intend to introduce family support, a key family situational resource, to investigate its moderating effect on the resource gain and loss mechanism of work connectivity behaviors.

Overall, we constructed a model to test how and when proactive work connectivity behaviors and passive work connectivity behaviors affect employee family harmony. This study makes three theory contributions. First, this study not only enriches the research on the attributes of working connectivity behaviors, but also explores the relationship between work connectivity behaviors and family harmony from the perspective of employees' subjective motivation. Second, this study examines the suppressing effect of self-efficacy and the mediating effect of ego depletion, that is, proactive work connectivity behaviors indirectly relate to family harmony through self-efficacy and passive work connectivity behaviors indirectly relate to family harmony through ego depletion. Third, this study reveals the contextual conditions under which work connectivity behaviors generate resource gains or losses, namely the moderating effect of family support.

## Theory and hypotheses

### Proactive/passive work connectivity behaviors and family harmony

Work connectivity behaviors refer to the behaviors that employees use mobile communication devices (cell phones, computers, etc.) to participate in work or contact with colleagues during non-working hours (Reinke and Ohly, 2021; Ren et al., 2021). Previous studies have classified work connectivity behaviors a priori, from the perspective of job characteristics and considered that work connectivity behaviors are either a kind of incentive job resources or a kind of stressful job demands, which may have a “double-edged sword” effect on employees' personal life and work (Ter Hoeven et al., 2016; Wan et al., 2019; Ren et al., 2021). Recent studies have pointed out that the way employees treat work will affect the impact of work on individuals (Schulte-Braucks et al., 2019). Therefore, as a typical boundary crossing behavior between work and non-work, how work connectivity behaviors will affect employees and their families depends on how employees treat such behavior (Huo et al., 2022). Based on previous studies, we consider the willingness of employees to participate in work connectivity behaviors and further divide work connectivity behaviors into proactive connectivity behaviors and passive connectivity behaviors from the perspective of subjective motivation (Huo et al., 2022). Among them, proactive connectivity behaviors refer to employees' subjective recognition and voluntary acceptance of handling work-related matters during non-working hours, while passive connectivity behaviors refer to that employees are required by the organization (such as leaders) to deal with work-related matters during non-working hours, which means employee involuntary and controlled behavior responding to leaders or colleagues during non-work hours (Piazza, 2007; Ohly and Latour, 2014; Huo et al., 2022). Therefore, whether work connectivity behaviors can play a positive role depends on the employees' work connectivity willingness, which may have a differential impact on the gain and loss of employees' resources.

On the one hand, proactive connectivity behaviors can be used as the job resource to effectively promote the accumulation of individual resources in the work field, and guide the infiltration and transfer of job resources to the family field to promote the performance of individual family roles, so as to achieve family harmony (Mazmanian et al., 2013; Derks et al., 2016; Ma et al., 2016; Carvalho et al., 2021; Reinke and Ohly, 2021). Specifically, employees will actively participate in work connectivity behaviors under the motivation of autonomy, and hope to meet their needs in autonomy, competence and relatedness (Ohly and Latour, 2014). The resources generated in the process of meeting needs can effectively spill over to the family field, thus promoting family harmony. First of all, work connectivity behavior is a specific behavior formed with the development of communication technology, which can be seen as a product of a special new work situation pattern. It breaks the time and space constraints of work, which can make the office space not limited to office buildings, and the working hours no longer limited to fixed working hours (Schlachter et al., 2018). When employees voluntarily choose to use mobile communication devices to work in non-working hours, mobile communication devices can give employees more flexibility and autonomy in work, provide them with more space to design the content and process of work tasks independently, improve their sense of freedom and control of work to meet their own needs for autonomy (Fujimoto et al., 2016). Secondly, proactive connectivity behaviors reflect employees' active self-dedication and extra efforts at work, which can help employees accumulate knowledge and skills at work, ultimately achieve their work goals and improve their work ability to meet their self-worth realization and competence needs (Carvalho et al., 2021). Finally, employees' active participation in work connectivity behaviors can bring them closer to their colleagues and organizations and achieve relational interaction with others, thus meeting their relatedness needs (Ohly and Latour, 2014). The above activities can effectively meet the employees' three basic psychological needs of autonomy, competence and relatedness, so as to obtain positive emotions, personal efficacy, happiness and other positive psychological states, which will help employees handle family affairs with a more optimistic attitude. Furthermore, positive emotions can be transmitted and shared among family members through empathy mechanism, which enhances the affection among family members and is conducive to family harmony (Reinke and Ohly, 2021).

On the other hand, proactive work connectivity behaviors and passive work connectivity behaviors can also be seen as job demands, which urge employees to deal with work-related issues at home. These behaviors objectively increase the working hours and workload of employees, consume the time and energy that employees would have invested in their families, hinder them from fulfilling their family responsibilities, and ultimately lead to complaints and dissatisfaction from other family members, which is detrimental to family harmony (Boswell and Olson-Buchanan, 2007). In addition, we further propose that compared with proactive work connectivity behaviors, passive work connectivity behaviors have a stronger negative impact on family harmony. One qualitative study showed that employees who were forced to engage in work connectivity behaviors after work reported more bad experiences than those who volunteered to do so (Khalid et al., 2021). When employees voluntarily choose to work with mobile communication devices after hours, their sense of control over work will promote employees to handle work more efficiently, which is conducive to achieving their work goals, thus ensuring a normal psychological detachment process, making them easier to recover from work and reducing the occupation of time and resources in the family field (Ohly and Latour, 2014; Reinke and Ohly, 2021). On the contrary, if employees are forced to participate in work connectivity behaviors due to external pressure, they are always under pressure, which makes it more difficult to recover from their work state and leads to continuous depletion of their personal resources (Lee et al., 2021). In this case, employees are unable to engage in family affairs due to extreme physical and mental exhaustion. Furthermore, employees are more likely to have negative emotions such as anxiety and irritability, which will be conveyed to other family members through mutual empathy in the family field, thus causing greater harm to family harmony (Sonnentag, 2018).

In conclusion, proactive work connectivity behaviors may have both positive and negative effects on family harmony. From the perspective of job resource spillover, proactive work connectivity behaviors have a positive impact on family harmony. From the perspective of job demands, proactive work connectivity behaviors have a negative impact on family harmony. Due to the positive and negative relationship between them, we do not propose a one-way impact hypothesis. Passive work connectivity behaviors have only a negative effect on family harmony. Therefore, the following hypotheses are proposed.

H1: Proactive work connectivity behaviors will have a significant impact on family harmony.
H2a: Passive work connectivity behaviors will have a negative impact on family harmony.
H2b: Compared with proactive work connectivity behaviors, passive work connectivity behaviors will have a stronger negative impact on family harmony.

### The mediating role of self-efficacy

Proactive work connectivity behaviors can improve employees' self-efficacy through resource generation functions. Self-efficacy is a degree of confidence in one's ability, which is expressed in the extent to which an individual believes that he or she can successfully perform tasks and achieve expected results (Bandura, 1986). For employees, self-efficacy is an important personal resource (Carvalho et al., 2021). Moreover, rich job resources are an important way to generate personal resources. When employees voluntarily deal with work affairs during non-working hours, they can obtain great work autonomy (job resources), such as free choice of working time and workplace, thus enhancing their sense of control over work (Richardson and Benbunan-Fich, 2011). Work efficiency will also be improved to a certain extent, which will help employees achieve their work goals. In this process, employees will gain more self-efficacy (personal resources) at work (Carvalho et al., 2021; Huo et al., 2022).

According to the W-HR model, positive family outcomes will occur when resources in the work domain increase personal resources and are used to improve family life (ten Brummelhuis and Bakker, 2012). Thus, the self-efficacy (personal resources) obtained by employees at work can produce positive spillover effects on family harmony (Carvalho et al., 2021). Family harmony refers to forbearance, effective communication, conflict resolution, family identity, and quality time with family. It is often expressed as a relationship of intimacy, harmony, happiness, cooperation, and mutual identity, and is considered to be the source of family happiness (Kavikondala et al., 2016). The personal resources accumulated in the positive work experience of employees can help them better perform their family duties, which is conducive to family harmony (Greenhaus and Powell, 2006). First of all, as a positive psychological resource, self-efficacy can stimulate employees' work motivation, enable them to obtain positive emotions at work, and maintain a high energy level (Judge and Bono, 2001). When employees have positive emotions, it can promote their initiative to stay close to family members, more likely to notice the various needs of family members, and consciously perform family-related roles and responsibilities, which is conducive to effective communication between family members as well as the establishment of friendly and interactive relationships (Watson et al., 1999), and thus promote family harmony. Secondly, self-efficacy can help employees adjust their cognition and actions, such as being confident in the face of family problems, believing that they can overcome the problems, and being willing to work hard for them (McNatt and Judge, 2008). Moreover, it can also encourage employees to come up with more ways to solve contradictions and conflicts when faced with complicated family matters, leading them to experience family harmony. In conclusion, proactive work connectivity behaviors can increase employees' self-efficacy, thereby promoting family harmony. Therefore, the following hypothesis is proposed.

**Hypothesis 3:** Self-efficacy will mediate the relationship between proactive work connectivity behaviors and family harmony.

### The mediation role of ego depletion

Passive work connectivity behaviors lead to employee ego depletion through a resource loss mechanism. Ego depletion refers to the state in which employees' psychological resources are exhausted after a period of self-regulation activities (Hagger et al., 2010). When employees are forced to participate in work connectivity behaviors, it means that employees are coerced to stay on call anytime and anywhere, which implies higher expectations of the organization for employees' working hours and intensity, thus increasing the perceived work pressure and role load (job demands) of employees (Huo et al., 2022). Under high work pressure, employees will put more effort and invest more time, energy, and emotional resources than under non-high work pressure, which will accelerate the loss of emotional, cognitive, and other psychological resources, and easily lead to ego depletion (Kang and Peng, 2019).

Based on the W-HR model, when the requirements of the work field consume personal resources and prevent individuals from contributing to the family field, it will lead to negative family outcomes (ten Brummelhuis and Bakker, 2012). As a result, employees suffer from ego depletion at work, which leads to negative spillover effects and adverse effects on family harmony. To be specific, when employees are forced to participate in work connectivity behaviors and suffer from ego depletion, they will lack sufficient resources to fulfill their family responsibilities, which harms family harmony. Firstly, the psychological resources possessed by individuals are limited, and the depletion of self-regulation activities in the work field will reduce the available resources for self-regulation activities in the family field (Tangney et al., 2004; Lee et al., 2021). In the case of ego depletion, employees lack enough time and energy to accompany their families and pay attention to the needs of family members, which is more likely to create family conflicts and is not conducive to family harmony. At the same time, employees who suffer from ego depletion will feel exhausted, and it is difficult to obtain a good work experience and experience the positive spillover effect between work and family (Greenhaus and Powell, 2006; Wan et al., 2019). Secondly, when employees lose resources due to work, they may bring the bad state at work into the family field, such as anger, depression, anxiety, and other bad emotions generated at work, which are easy to cause interpersonal harm to the family (Tang et al., 2014; Xie et al., 2018). In addition, in a bad state of ego depletion, employees will reduce their willingness and motivation to participate in family activities and perform family duties, which is also harmful to family harmony (Greenhaus and Powell, 2006). To sum up, passive connectivity behaviors can lead to employee ego depletion, and then reduce family harmony. Therefore, the following hypothesis is proposed.

**Hypothesis 4:** Ego depletion will mediate the relationship between passive work connectivity behaviors and family harmony.

### The moderating role of family support

Based on the W-HR model, situational resources are regarded as resource investments, which can effectively enhance the positive impact of job resources on individuals and alleviate the negative impact of job demands on personal resources (ten Brummelhuis and Bakker, 2012). Therefore, this study suggests that family support, as a situational resource, can not only enhance the relationship between proactive work connectivity behaviors and self-efficacy but also weaken the relationship between passive work connectivity behaviors and ego depletion. Family support refers to the care and helps that employees receive from family members (parents, partners, children) who help individuals better achieve work goals by providing instrumental advice and emotional resources (Siu et al., 2010). As a key resource, family support can not only be used as an initial resource to reduce resource loss but also as a new resource to generate greater resource increment (ten Brummelhuis and Bakker, 2012). On the one hand, family support can effectively promote the resource gain spiral. Specifically, with high-level family support, employees can get more emotional and instrumental support from their families. For example, family members can encourage employees to increase their confidence, listen patiently and give emotional care to employees when they are depressed, etc. (Chen and Ellis, 2021). This positive and pleasant family experience tends to bring pleasure and happiness to employees, and positive emotion can enhance the identification of individual's self-ability and the belief of producing more beneficial results (Lee and Shin, 2017), as well as increase their self-efficacy level at work. At this time, employees are more likely to focus on resource acquisition and pursue opportunities to acquire resources (Halbesleben et al., 2014). In other words, employees will regard proactive connectivity behaviors as an opportunity to obtain resources, which makes it easier to accumulate self-efficacy in their work. In addition, family members help employees to perform part of their family responsibilities so that employees can invest more time and energy to complete their work goals and overcome their work difficulties, which is also conducive to the accumulation of employees' self-efficacy. On the other hand, family support can effectively restrain the resource depletion spiral. A high level of family support provides resources for employees in the process of work connectivity behaviors, thereby alleviating or even avoiding ego depletion caused by passive connectivity behaviors. However, under the low level of family support, individuals are faced with limited resources and are prone to fall into the spiral of resource depletion. At this time, employees are more sensitive to resource loss and thus amplify their perception of ego depletion (Halbesleben et al., 2014). Therefore, the following hypothesis is proposed.

**Hypothesis 5:** Family support will moderate the relationship between proactive work connectivity behaviors and self-efficacy, such that proactive work connectivity behaviors will affect self-efficacy more positively with higher rather than lower levels of family support.
**Hypothesis 6:** Family support will moderate the relationship between passive work connectivity behaviors and ego depletion, passive work connectivity behaviors will affect ego depletion more positively with lower rather than higher levels of family support.

### The moderated mediating role of family support

Based on the W-HR model, situational resources can help employees effectively use work resources and cope with job demands, increase personal resources, and then benefit the outcome in the family domain (ten Brummelhuis and Bakker, 2012). Therefore, this study further constructed a moderated mediating effect model, that is, the influence of proactive connectivity behaviors on family harmony through self-efficacy, and the influence of passive connectivity behaviors on family harmony through ego depletion would be moderated by family support. Under the high level of family support, employees will take the proactive connectivity behaviors as an opportunity to obtain resources, gain more self-efficacy, and then produce positive spillovers to the family field to promote family harmony. On the contrary, under the low level of family support, employees will see passive connectivity behaviors as a threat to resources, amplify their perception of ego depletion, and then produce negative spillovers to the family field, which is harmful to family harmony. Therefore, the following hypothesis is proposed.

**Hypothesis 7:** Family support will moderate the indirect effect of proactive work connectivity behaviors on family harmony through self-efficacy, such that the indirect effect will be more positive with a high level of family support.
**Hypothesis 8:** Family support will moderate the indirect effect of passive work connectivity behaviors on family harmony through ego depletion, such that the mediating effect will be less negative with a high level of family support.

**Figure 1:**
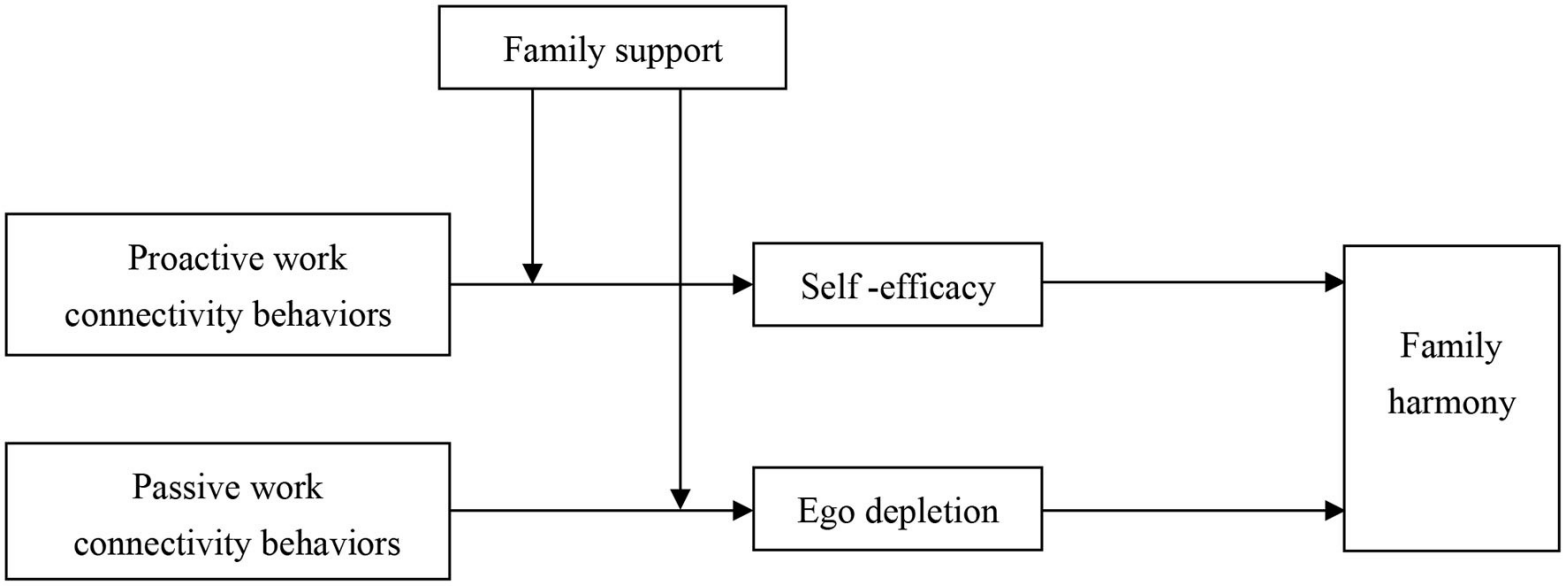
The theoretical model.

The theoretical model of this study is shown in Figure 1.

## Materials and methods

### Sample and procedure

In this study, data were collected through an online questionnaire survey, involving the employees with fixed working hours in the Internet, e-commerce, finance, software, information technology and other industries in Wuhan, Changsha, and Shenzhen from China, because the use of mobile communication devices is typically important for them to carry out their job, making them more representative in terms of contemporary employees' work connectivity behaviors and work-family Interaction. To avoid common method bias, this study adopts a multi-time point data collection method. In the first wave, the human resources supervisor of each enterprise in the sample was contacted by the assistant investigator, and the link to the electronic questionnaire was sent to them and the procedure and purpose of the survey were explained. Then, the questionnaire was distributed to the employees of the enterprise. During the distribution, it is emphasized repeatedly that the required content is for academic research only and is completely anonymous to ensure that the participants can answer truthfully according to their circumstances. The first questionnaire included demographic variables such as gender, tenure, education level, marriage, and fertility status, as well as proactive work connectivity behaviors, passive work connectivity behaviors, and family support variables. Finally, we received 426 valid samples. A second survey was conducted 2 weeks later, the electronic link was also sent to the human resources supervisor who was contacted before, and then the human resources supervisor send the questionnaire link to the previous sample, including demographic variables as well as variables of self-efficacy, and ego depletion. Finally, we received 380 valid samples. A third survey was conducted 2 weeks later, the electronic link was also sent to the human resources supervisor who was contacted before, and then the human resources supervisor send the questionnaire link to the previous sample, including demographic variables as well as variables of family harmony. To match the data of the three surveys, the participants were asked to fill in the last four digits of their mobile phone numbers at the end of each questionnaire. After all the data collection, we ultimately obtained 364 valid questionnaires. The descriptive characteristics of the samples are shown in Table 1.

**Table 1:** Descriptive characteristics of samples (*n* = 364).

**Characteristic**	**Category**	**Number**	**Percentage (%)**
Gender	Male	194	53.3%
	Female	170	46.7%
Tenure	5 years or below	134	36.8%
	5–10 years	74	20.3%
	10 years or above	156	42.9%
Education	Junior college or below	73	20.1%
	Bachelor degree	240	65.9%
	Master degree	51	14%
Marriage	Married	224	61.5%
	Not married	140	38.5%
Fertility	At least one child	213	58.5%
	No children	151	41.5%

### Measures

In order to ensure the reliability and validity of the questionnaire measurement, all variables in this study were measured by confirmed mature scale, and we strictly followed the standard translation and back-translation procedure. Furthermore, we invited six enterprise employees to form a focus group and test the content validity of questionnaire items. 80% of employees can understand the meaning of the items, ensuring the requirements of content validity. All scale items in this study were measured using a 5-point Likert scale.

### Proactive/passive work connectivity behaviors

Proactive/Passive work connectivity behaviors (PR-WCB/PA-WCB) were measured with the six-item scale developed by Fenner and Renn (2010). On the basis of the original question items, we applied it to different proactive and passive scenarios to reflect the proactively and passivity of work connectivity behaviors. And the clear definitions of proactive and passive connected behaviors were written at the beginning of the questionnaire to ensure that participants could accurately understand the meaning of the item. A sample item of Proactive work connectivity behaviors is “When I fall behind in my work during the day, I proactively work hard at home at night or on weekends to get caught up by using my cell phone” (1 = “never”, 5 = “always”). The Cronbach's alpha for this scale was 0.852. A sample item of Passive work connectivity behaviors is “when I return home from work, I passively use my cell phone or computer for work-related tasks” (1 = “never”, 5 = “always”). The Cronbach's alpha for this scale was 0.817.

### Self-efficacy

Self-efficacy (SE) was measured with the ten-item scale developed by Schwarzer et al. (1997a,b). A sample is “I can always manage to solve difficult problems if I try hard enough” (1 = “completely disagree”, 5 = “completely agree”). The Cronbach's alpha for this scale was 0.905.

### Ego depletion

Ego depletion (ED) was measured with the five-item scale developed by Lin and Johnson (2015). A sample is “I feel like my willpower is gone” (1 = “completely disagree”, 5 = “completely agree”). The Cronbach's alpha for this scale was 0.873.

### Family support

Family support (FS) was measured with the ten-item scale developed by Chen and Ellis (2021). A sample is “how much the family members provide you with encouragement” (1 = “not at all”, 5 = “a great deal”). The Cronbach's alpha for this scale was 0.917.

### Family harmony

Family harmony (FH) was measured with the five-item scale developed by Schwarzer et al. (1997a,b). A sample is “My family is harmonious” (1 = “completely disagree”, 5 = “completely agree”). The Cronbach's alpha for this scale was 0.869.

### Control variables

To minimize the estimation bias caused by missing variables, we controlled the demographic variables of gender, tenure, education, marriage, and fertility status. In this study based on the previous literatures (Boswell and Olson-Buchanan, 2007; Richardson and Thompson, 2012; Dumas and Perry-Smith, 2018; Xie et al., 2018; Yang et al., 2022).

## Analysis and results

### Confirmatory factor analysis

To better verify the discriminant validity of each variable in the research model, we used Mplus8 to conduct confirmatory factor analysis (CFA). To be specific, constructed a six-factor model including proactive work connectivity behaviors, passive work connectivity behaviors, self-efficacy, ego depletion, family support, and family harmony. The results of confirmatory factor analysis (Table 2) showed that the fitting effect of the six-factor model (χ^2^ = 1,332.474, df = 804, χ^2^/df = 1.657, RMSEA = 0.042, CFI = 0.929, TLI = 0.924) was significantly better than that of other competitive models. It indicates that the variables in this study have good discriminative validity, which lays a foundation for subsequent analysis.

**Table 2:** Results for confirmatory factor analysis.

**Model**	**χ^2^**	**df**	**χ^2^/df**	**RMSEA**	**CFI**	**TLI**
Six factors: PR-WCB; PC-WCB SE; ED; FS; FH	1,332.474	804	1.657	0.042	0.929	0.924
Five factors: PR-WCB+PC-WCB; SE; ED; FS; FH	2,056.363	809	2.542	0.065	0.832	0.821
Four factors: PR-WCB+PC-WCB; SE; ED; FS+FH	2,956.724	813	3.637	0.085	0.711	0.694
Three factors: PR-WCB+PC-WCB; SE+ED; FS+FH	3,787.935	816	4.642	0.100	0.600	0.578
One factor: PR-WCB+PC-WCB +SE+ED+FS+FH	5,100.073	819	6.227	0.120	0.423	0.394

N = 364.

PR-WCB, proactive work connectivity behaviors; PC-WCB, passive work connectivity behaviors; SE, self-efficacy; ED, ego depletion; FS, family support; FH, family harmony.

Table 3 shows factor loadings, average variance extracted (AVE) and the composite reliability (CR). According to Fornell and Larcker (1981), CR should exceed 0.6, and AVE should exceed 0.5 under ideal condition, while 0.36–0.5 are acceptable. Hence, all items for convergent validity were met.

**Table 3:** Composite reliability and convergent validity.

**Variables**	**No. of items**	**Loadings range**	**AVE**	**CR**
Proactive work connectivity behaviors	6	[0.694–0.724]	0.491	0.852
Passive work connectivity behaviors	6	[0.642–0.671]	0.427	0.817
Self-efficacy	10	[0.666–0.745]	0.488	0.905
Ego depletion	5	[0.743–0.786]	0.579	0.873
Family support	10	[0.694–0.756]	0.524	0.917
Family harmony	5	[0.681–0.935]	0.589	0.876

### Common method variance

To reduce the common method bias in the process of data collection, this study adopts a multi-time point method to obtain the research data. Harman single factor test was used to test the common method deviation. The results show that the first factor only explains 27.159% of the total variance, which is far less than the critical value of 40%. Therefore, the common method bias in this study is not serious and has little impact on the results. However, considering the insensitivity of the Harman single-factor test, we conducted a latent method factor based on the six-factor model to test CMV. The analysis results showed that the seven-factor model after the addition of the latent method factor (χ^2^ = 1,328.559, df = 803, χ^2^/df = 1.654, RMSEA = 0.042, CFI = 0.929, TLI = 0.924) was not significantly better than the six-factor model, indicating that our study does not have serious common method biases (Podsakoff et al., 2003).

### Descriptive analysis and correlation analysis

Table 4 presents the means, standard deviations, and correlation coefficients of the main variables in this study. As shown in Table 4, proactive work connectivity behaviors were positively related to self-efficacy (r = 0.241, *p* < 0.01). Passive work connectivity behaviors were positively related to ego depletion (r = 0.484, *p* < 0.01). Self-efficacy was positively related to family harmony (r = 0.292, *p* < 0.01). Ego depletion was negatively related to family harmony (r = −0.436, *p* < 0.01), which provided a preliminary test of the study hypothesis.

**Table 4:** Means, standard deviations and correlations among variables.

**Variables**	**1**	**2**	**3**	**4**	**5**	**6**	**7**	**8**	**9**	**10**	**11**
1. Gender	1.000										
2. Tenure	0.070	1.000									
3. Education	−0.041	−0.237^**^	1.000								
4. Marriage	0.007	0.643^**^	−0.199^**^	1.000							
5. Children	−0.071	0.710^**^	−0.233^**^	0.830^**^	1.000						
6. PR-WCB	0.042	0.082	0.001	−0.011	0.008	1.000					
7. PA-WCB	0.073	−0.048	−0.008	0.020	−0.051	0.113^*^	1.000				
8. SE	0.006	0.075	−0.077	0.084	0.124^*^	0.241^**^	−0.352^**^	1.000			
9. ED	0.037	0.029	−0.034	−0.022	−0.043	0.256^**^	0.484^**^	−0.244^**^	1.000		
10.FH	−0.049	−0.081	0.015	−0.033	−0.085	−0.285^**^	−0.365^**^	0.292^**^	−0.436^**^	1.000	
11. FS	0.052	0.004	0.010	−0.047	−0.023	0.027	−0.490^**^	0.358^**^	−0.354^**^	0.264^**^	1.000
Mean	0.530	2.060	1.940	0.620	0.590	3.800	2.961	3.398	3.177	2.875	3.043
SD	0.500	0.892	0.581	0.487	0.493	0.700	0.690	0.727	0.794	0.766	0.756

N = 364.

* p < 0.05,

** p < 0.01. Gender: male = 1, female = 0; Tenure: 5 years or below = 1, 5–10 years = 2, 10 years or above = 3; Education: junior college or below = 1, bachelor degree = 2, master degree = 3; Marriage: Married = 1, Not married = 0; Children: at least one child = 1, no children = 0.

PR-WCB, proactive work connectivity behaviors; PA-WCB, passive work connectivity behaviors; SE, self-efficacy; ED, ego depletion; FS, family support; FH, family harmony.

### Hypothesis testing

Research hypotheses were tested using hierarchical regression analysis and bootstrapping in SPSS 26.0, and the results were shown in Table 5.

**Table 5:** Results for regression analysis.

**Model**	**1**	**2**	**3**	**4**	**5**	**6**	**7**	**8**	**9**	**10**	**11**
**Variables**	**SE**			**ED**			**FH**				
Gender	0.020	0.011	−0.025	0.020	−0.011	0.001	−0.067	−0.056	−0.060	−0.043	−0.046
Tenure	−0.044	−0.076	−0.061	0.106	0.131	0.080	−0.007	0.030	0.060	−0.027	0.017
Education	−0.052	−0.057	−0.071	−0.036	−0.026	−0.050	−0.014	−0.009	0.014	−0.023	−0.031
Marriage	−0.067	−0.034	0.023	0.006	−0.095	−0.087	0.192	0.154	0.167	0.271^**^	0.239^**^
Children	0.200	0.187	0.207	−0.122	−0.031	−0.102	−0.263^*^	−0.248^*^	−0.323^*^	−0.334^**^	−0.345^**^
PR-WCB		0.241^***^	0.274^***^					−0.276^***^	−0.373^***^		
PA-WCB					0.491^***^	0.472^***^				−0.385^***^	−0.221^***^
SE									0.404^***^		
ED											−0.334^***^
FS			0.339^***^			−0.191^***^					
FS^*^PR-WCB			0.182^**^								
FS^*^PA-WCB						−0.332^***^					
R^2^	0.019	0.076	0.224	0.009	0.245	0.306	0.023	0.098	0.249	0.168	0.252
Δ*R*^2^	0.019	0.057	0.022	0.009	0.236	0.043	0.023	0.075	0.150	0.145	0.084
F	1.420	4.927^***^	12.800^***^	0.623	19.299^***^	19.561^***^	1.690	6.479^***^	16.828^***^	12.029^***^	17.171^***^

N = 364.

* p < 0.05,

** p < 0.01,

*** p < 0.001.

PR-WCB, proactive work connectivity behaviors; PA-WCB, passive work connectivity behaviors; SE, self-efficacy; ED, ego depletion; FS, family support; FH, family harmony.

Analysis of the main effects of proactive work connectivity behaviors and passive work connectivity behaviors on family harmony. In model 8, proactive work connectivity behaviors had a significant negative impact on family harmony (β = −0.276, *p* < 0.001). Thus, H1 was supported. In model 10, passive work connectivity behaviors had a significant negative impact on family harmony (β = −0.385, *p* < 0.001). Compared with proactive work connectivity behaviors (β = −0.276), passive work connectivity behaviors (β = −0.385) have a stronger negative impact on family harmony. Thus, H2a and H2b were supported.

Analysis of the mediating effects of self-efficacy and ego depletion. Model 2 showed that proactive work connectivity behaviors had a significant positive impact on self-efficacy (β = 0.241, *p* < 0.001). According to model 9, when proactive work connectivity behaviors and self-efficacy are included in the regression equation at the same time to predict family harmony, the regression coefficient of proactive work connectivity behaviors and family harmony is still significant (β = −0.373, *p* < 0.001), at the same time, self-efficacy had a significant positive impact on family harmony (β = 0.404, *p* < 0.001). Thus, H3 was supported.

Model 5 showed that passive work connectivity behaviors had a significant positive impact on ego depletion (β = 0.491, *p* < 0.001). According to model 11, when passive work connectivity behaviors and ego-depletion are included in the regression equation at the same time to predict family harmony, the regression coefficient of proactive work connectivity behaviors and family harmony is still significant (β = −0.221, *p* < 0.001), at the same time, ego depletion had a significant negative impact on family harmony (β = −0.334, *p* < 0.001). Thus, H4 was supported.

Further, using the SPSS macro program PROCESS' MODEL4 proposed by Preacher and Hayes (2008) to analyze the indirect effect of self-efficacy and ego depletion. The bootstrapping sample size was set to 5,000, the confidence interval was set to 95%, and the results were shown in Table 6. The indirect effect of self-efficacy between proactive work connectivity behaviors and family harmony was 0.106 and the 95% confidence interval (LLCI = 0.056, ULCI = 0.161) did not include 0, indicating that Hypothesis 3 got fully supported. However, we further found that the total effect (β = −0.303, 95%CI = [−0.412, −0,194]) and the direct effect (β = −0.409, 95%CI = [−0.512, −0.306]) of proactive connectivity behaviors on family harmony were negative. The indirect effect of self-efficacy between proactive connectivity behaviors and family harmony was positive, that is, the sign of the direct effect coefficient was opposite to that of the indirect effect coefficient, indicating that self-efficacy played a suppressing effect between proactive connectivity behaviors and family harmony (MacKinnon et al., 2000; Wen and Ye, 2014). The indirect effect of ego depletion between passive work connectivity behavior and family harmony was −0.182 and the 95% confidence interval (LLCI = −0.275, ULCI = −0.106) did not include 0, indicating that Hypothesis 4 got fully supported.

**Table 6:** Results of bootstrapping mediation effect examination.

**Effect**	**Estimate**	**S.E**.	**95%LLCI**	**95%ULCI**
**Proactive work connectivity behaviors**→**self-efficacy**→**family harmony**				
Total effect	−0.303	0.056	−0.412	−0.194
Direct effect	−0.409	0.052	−0.512	−0.306
Indirect effect	0.106	0.027	0.056	0.161
**Passive work connectivity behaviors**→**ego depletion**→**family harmony**				
Total effect	−0.427	0.054	−0.533	−0.320
Direct effect	−0.245	0.059	−0.361	−0.129
Indirect effect	−0.182	0.043	−0.275	−0.106

**Figure 2:**
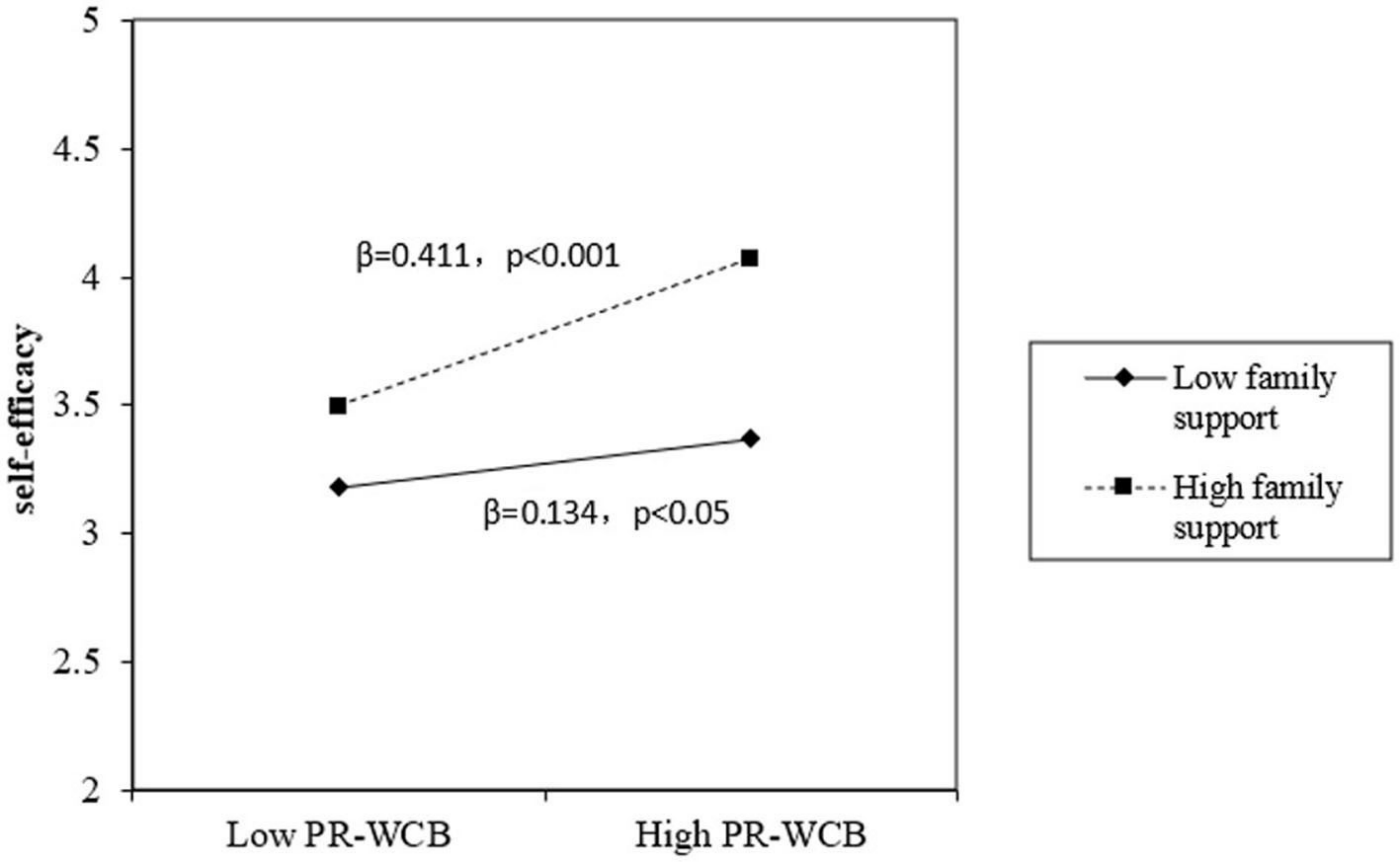
The moderating effect of family support on the impact of PR-WCB on self-efficacy.

**Figure 3:**
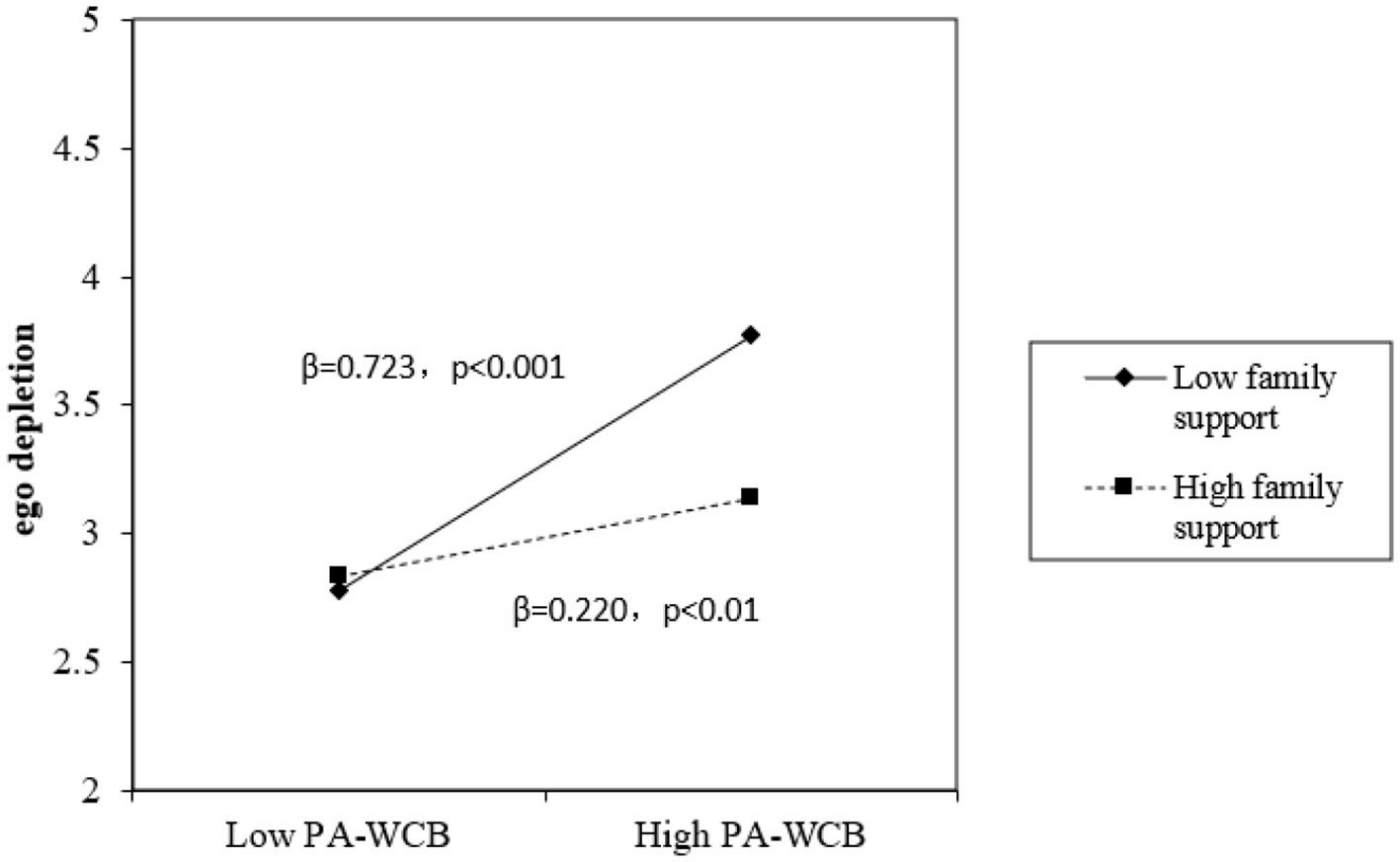
The moderating effect of family support on the impact of PA-WCB on ego depletion.

Furthermore, we analyzed the interactive effects of proactive work connectivity behaviors and passive work connectivity behaviors with family support. In model 3, the results indicate that the interaction between proactive work connectivity behaviors with family support was significantly related to self-efficacy (β = 0.182, *p* < 0.01), thus Hypothesis 3 was supported. In model 6, the results indicate that the interaction between passive work connectivity behaviors with family support was significantly related to ego depletion (β = −0.332, *p* < 0.001), thus Hypothesis 4 was supported. We also adopted simple slope analysis to describe the difference in the impact of proactive work connectivity behaviors on self-efficacy with different levels of family support, which were based on one standard deviation above and below the mean (±1 SD). As plotted in Figure 2, when family support was at a high level (+1 SD), the positive impact of proactive work connectivity behaviors on self-efficacy was stronger (β = 0.411, *p* < 0.001). On the contrary, when the family support was at a low level (−1 SD), the positive impact of proactive work connectivity behaviors on self-efficacy was weaker (β = 0.136, *p* < 0.05). Thus, further supporting Hypothesis 5. Similarly, as plotted in Figure 3, when family support was at a high level (+1 SD), the positive impact of passive work connectivity behaviors on ego depletion was weaker (β = 0.220, *p* < 0.01). On the contrary, when the family support was at a low level (−1 SD), the positive impact of passive work connectivity behaviors on ego depletion was stronger (β = 0.723, *p* < 0.001). Thus, further supporting Hypothesis 6.

Finally, we used PROCESS' MODEL7 to examine the whole moderated mediation model, and the results were shown in Table 7. The results indicate that the indirect effect of proactive work connectivity behaviors on family harmony through self-efficacy (β = 0.175, 95%CI = [0.101, 0.252]) was stronger with a high level of family support. In contrast, the indirect effect (β = 0.058, 95%CI = [0.003, 0.114]) was weaker when the family support was at a low level. And the differential effect between high levels and low levels of family support was significant (β = 0.117, 95%CI = [0.037, 0.204). Thus, Hypothesis 7 was supported. Similarly, the results indicate that the indirect effect of passive work connectivity behaviors on family harmony through ego depletion (β = −0,233, 95%CI = [−0.368, −0.134]) was stronger with a low level of family support. In contrast, the indirect effect (β = −0.071, 95%CI = [−0.137, −0.018]) was weaker when the family support was at a high level. And the differential effect between high levels and low levels of family support was significant (β = 0.162, 95%CI = [0.074, 0.280). Thus, Hypothesis 8 was supported.

**Table 7:** Results for moderated mediation effect.

**Effect**	**Estimate**	**S.E**.	**95%LLCI**	**95%ULCI**
**Proactive work connectivity behaviors**→**self-efficacy**→**family harmony**				
Moderated mediation	0.077	0.028	0.024	0.135
Low level of family support	0.058	0.029	0.003	0.114
High level of family support	0.175	0.038	0.101	0.252
Difference	0.117	0.042	0.037	0.204
**Passive work connectivity behaviors**→**ego depletion**→**family harmony**				
Moderated mediation	0.107	0.035	0.049	0.185
Low level of family support	−0.233	0.060	−0.368	−0.134
High level of family support	−0.071	0.030	−0.137	−0.018
Difference	0.162	0.053	0.074	0.280

### Supplementary analyses: Multiple mediating effects test

The existence of the “suppressing effect” of self-efficacy, on the one hand, proved the existence of the indirect mechanism that proactive work connectivity behaviors affected family harmony through self-efficacy, on the other hand, it also showed that there were more effective mediators between proactive work connectivity behaviors and family harmony (Kenny et al., 2003). Therefore, we included self-efficacy and ego depletion into the model at the same time to explore whether self-efficacy and ego depletion play multiple mediating roles between proactive work connectivity behaviors and family harmony (as shown in Table 8). The results showed that proactive work connectivity behaviors have a positive impact on self-efficacy (β = 0.251, 95%CI = [0.146, 0.356]) and ego depletion (β = 0.289, 95%CI = [0.174 0.404]), which indicated that proactive work connectivity behaviors had both gain and loss effects on personal resources of employees (self-efficacy and ego depletion). The indirect effect of ego depletion between proactive work connectivity behaviors and family harmony was −0.083 and the 95% confidence interval (LLCI = −0.139, ULCI = −0.039) did not include 0, indicating that ego depletion mediates the relationship between proactive work connectivity behaviors and family harmony. Furthermore, we compared the mediating effects of self-efficacy and ego depletion. The mediating effect coefficient of self-efficacy (β = 0.081, 95%CI = [0.040, 0.130]) was a little less than that of ego depletion (β = −0.083, 95%CI = [−0.139, −0.039]). The difference in coefficient between the two mediating effects was 0.164 and the 95% confidence interval (LLCI = −0.111, ULCI = 0.226) did not include 0.

**Table 8:** Results of multiple mediating effects test.

**Effect**	**Estimate**	**S.E**	**95%LLCI**	**95%ULCI**
Total effect PR-WCB → FH	−0.303	0.056	−0.412	−0.194
Direct effect PR-WCB → FH	−0.300	0.053	−0.405	−0.197
Direct effect PR-WCB → SE	0.251	0.053	0.146	0.356
Direct effect PR-WCB → ED	0.289	0.058	0.174	0.404
Direct effect SE → FH	0.323	0.051	0.223	0.422
Direct effect ED → FH	−0.287	0.046	−0.378	−0.196
Indirect effect1 PR-WCB → SE → FH	0.081	0.023	0.040	0.130
Indirect effect2 PR-WCB → ED → FH	−0.083	0.026	−0.139	−0.039
IND1 + IND2	−0.002	0.039	−0.800	0.075
IND1 - IND2	0.164	0.029	0.111	0.226

PR-WCB, proactive work connectivity behaviors; SE, self-efficacy; ED, ego depletion; FH, family harmony.

IND1 + IND2 = the mediating effect of Self-efficacy plus the mediating effect of ego depletion. IND1 - IND2 = the mediating effect of self-efficacy subtract the mediating effect of ego depletion.

## Discussion

From the work-home resources model perspective, we propose a theoretical model that proactive work connectivity behaviors and Passive work connectivity behaviors impact family harmony through self-efficacy and ego depletion, and we explore the moderating role of family support in this relationship. Based on survey data collected from 364 questionnaires by using a three-wave time-lagged design, we get the following conclusions.

First, we tested the main effect between proactive/passive work connectivity behaviors and family harmony. The results showed that both proactive work connectivity behaviors and passive work connectivity behaviors have a significant negative impact on family harmony. However, compared with proactive connectivity behaviors, passive connectivity behaviors are more harmful to family harmony.

Second, we tested the suppressing effect of self-efficacy. When employees actively participate in work connectivity behaviors, they can gain a sense of control over their work and effectively complete their work goals, thereby improving self-efficacy (Schaufeli and Taris, 2014), and the accumulated personal resources will actively spill over into the family field, which is beneficial to family harmony. However, the direct effect between proactive work connectivity behaviors and family harmony is negative, and proactive work connectivity has a significant positive impact on self-efficacy and ego depletion, which indicates that proactive work connectivity has both gain and loss effects on personal resources. Based on the three-dimensional model of job demands and resources, job related factors can be divided into three categories according to their impact on personal resources: job resources, challenge job demands and hindrance job demands. Among them, job resources can bring gains to personal resources, hindrance job demands will consume personal resources, and challenge job demands will both gain and consume personal resources (Crawford et al., 2010). Therefore, different from previous studies, work connectivity behaviors are classified into job resources and job demands a priori. This study considers that proactive work connectivity behaviors have the attribute of challenge job demands, while passive work connectivity behaviors have the attribute of hindrance job demands.

Third, we tested the mediating effect of ego depletion. When employees are forced to participate in work connectivity behaviors, the workload and work intensity will increase, making it impossible for employees to recover physically and mentally, which will lead to the continuous reduction of personal resources, resulting in a bad state of ego depletion. The bad emotions associated with this state will negatively spill over to the family field, which will be harmful to family harmony. This is consistent with the previous results of regarding work connectivity behaviors as job demands and discussing its negative effects (Xie et al., 2018). Further subdivided, because passive work connectivity can only lead to the loss of personal resources, it has the property of hindrance job demands.

Fourth, we tested the moderating effect of family support. This study found that family support not only positively moderated the relationship between proactive work connectivity behaviors and self-efficacy, but also moderated the mediating role of self-efficacy between proactive work connectivity behaviors and family harmony. In addition, family support not only negatively moderated the relationship between passive work connectivity behaviors and ego depletion, but also moderated the mediating effect of ego depletion on the relationship between passive work connectivity behaviors and family harmony. It shows that family support, as a very important situational resource, can effectively promote the gain spiral of personal resources and restrain the loss spiral of personal resources. When employees receive a high level of family support, they will accumulate more self-efficacy when they voluntarily participate in work connectivity behaviors and then promote family harmony. In addition, if employees are forced to engage in work connectivity behaviors, family support can effectively slow down the loss of personal resources, alleviate their ego depletion, and thus reduce the adverse impact on family harmony.

### Theoretical contributions

First, this study explored the relationship between work connectivity behaviors and family harmony from the perspective of employees' subjective motivation and extends the application of the W-HR model in the field of work connectivity behaviors research. Previous studies have mainly classified work connectivity behavior a priori, defined it as job resources or job demands, and discussed the positive and negative effects on work or family places respectively (Richardson and Thompson, 2012; Ter Hoeven et al., 2016). However, few studies have classified work connectivity behaviors into proactive work connectivity behaviors and passive work connectivity behaviors from the perspective of personal subjective motivation. This classification responds to the initiative of scholars to distinguish the attributes of work connectivity behaviors in future research (Ohly and Latour, 2014) and expands the research scope of the impact of work connectivity behaviors on the family field. In addition, this study also explored the attributes of proactive and passive connectivity behaviors, as well as their differential effects on individual family harmony. The results show that proactive connectivity behaviors have both promoting and inhibiting effects, which have the attributes of challenge job demands. Passive connectivity behaviors only have an inhibiting effect, so it has the attribute of hindrance job demands. These findings enrich the research on the attributes of working connectivity behaviors.

Second, this study investigated the internal mechanism of the effect of work connectivity behaviors on family harmony from a process perspective to further verify the applicability of the W-HR model. The process perspective refers to the view of work-family conflict and work-family enrichment as the interactive process between the work and family domains. Specifically, work-family conflict represents a process in which “demands in the work domain consume personal resources, resulting in increased negative outcomes in the family domain”, and work-family enrichment represents a process in which “resources in the work domain develop personal resources and drive increased positive outcomes in the other family domain” (ten Brummelhuis and Bakker, 2012). From the process perspective, the whole variable relationship is highlighted as a work-family conflict process and a work-family enrichment process. However, the existing research mainly adopts the perspective of outcome view, that is, work-family conflict and work-family enrichment are regarded as the outcome variables in the family field, and it is believed that the demands or resources in the work field produce the results of work-family conflict and work-family enrichment in the family field through the response of individual resources. In other words, the outcome view is reflected in the presence of variables such as work-family conflict and work-family enrichment in the model, rather than specific results in the work or family field, such as family harmony. Therefore, based on the original view of the W-HR model, we conducted research from the perspective of the process view and discussed that the proactive work connectivity behaviors lead to the increase of employees' resources, and the accumulated self-efficacy actively overflows to the family field, thereby promoting family harmony, which reflects the work-family enrichment process in the W-HR model. In addition, the passive work connectivity behaviors lead to the loss of personal resources, which leads to the negative spillover of employee ego depletion to the family field, thereby inhibiting family harmony, which reflects the work-family conflict process in the W-HR model. The above findings further shed light on the black box between work connectivity behaviors and family harmony.

Third, this study revealed the contextual conditions under which work connectivity behaviors generate resource gains or losses, namely the moderating effect of family support. The results found that under a high level of family support, proactive work connectivity behaviors would enhance the positive effect (improve self-efficacy). With low levels of family support, passive work connectivity can enhance its negative effect (increasing ego depletion). This indicates that family support can promote the resource gain function of proactive work connectivity behavior and alleviate the resource loss function of passive work connectivity behavior. In addition, previous studies generally support the resource gain function of family support (Seiger and Wiese, 2009; ten Brummelhuis and Bakker, 2012; Park and Fritz, 2015). It can be seen that the findings of this study are consistent with previous studies.

### Practical implications

First, organizations need to pay attention to employees' willingness to work connectivity. Managers should be aware that forcing employees to participate in work connectivity behavior is inefficient and will cause the loss of personal resources of employees, which is detrimental to their physical and mental health and family harmony. The manager can control the work connectivity behaviors in a reasonable range and negotiate with the employees about the work connectivity time that is acceptable to both sides. For example, the manager can fix a certain period of time to discuss the work during non-working hours to reduce the interference in the life of the employees. In conclusion, organizations should not advocate or even force employees to use mobile communication devices to deal with work-related affairs after work. An organizational culture that promotes the use of mobile communication devices to deal with work-related affairs during non-working hours may lead to the highly normalized use of mobile communication devices after hours, resulting in an “always on, always connected” organizational atmosphere. It is not conducive for employees to recover from long-term depletion (Reinke and Ohly, 2021). Organizations can allow employees to use mobile communication devices to deal with work affairs after work, but do not expect it.

Second, employees need to rationally understand and use work connectivity tools. With the update in information technology and the intensification of enterprise competition, the demand for employees' work connectivity is becoming increasingly urgent. It is becoming more pervasive to use mobile communication devices for employees to deal with work affairs during non-working hours. Therefore, for employees themselves, they need to take the initiative to adapt to the changes in work situations and ways brought about by the development of science and technology promptly on time, effectively arrange the time, reasonably set the boundary between work and life, and reduce the adverse impact of work connectivity behaviors on life. At the same time, employees need to correctly use work connectivity tools, fully understand and give play to the positive aspects of work connectivity behavior, and constantly accumulate personal resources to achieve the goal of using technology for their purposes. In addition, employees should not regard work connectivity behavior as an obligation. When they are forced to participate in work connectivity behavior and bring harm to themselves and their families, they can use the right to disconnect, and do not necessarily need to deal with work affairs in non-working hours. The best approach is to actively communicate with leaders about their actual expectations and preferences for dealing with work affairs in non-working hours, so as to obtain the initiative to use mobile communication devices, and achieve diversified and autonomous use of mobile communication devices.

Third, both organizations and employees should attach importance to the demands of employees' families. For the organizations, they can formulate work-family balance policies to help employees meet work and family demands (Bardoel et al., 2014). For example, regulate the working time regulations and emphasize that the non-working time should be the real non-working time, that is, the non-working time should be used for other areas of employees' life, whether it is family activities or personal activities. And it is not necessary to increase the expectation of availability beyond working hours, help employees divide their work and personal life, and strive for the support of their family members. For individual employees, while actively seeking support from family members should also actively perform family duties to promote a positive feedback loop of family harmony and support from family members. Moreover, employees should be careful not to invest “excessive” time and energy in their work. Relevant studies have shown that workaholism often leads to work-family conflict, which is not conducive to family harmony (Daniel et al., 2022).

### Limitations and future research

First, this study unexpectedly found that proactive connectivity behaviors have both positive and negative effects, which has a “double-edged sword” effect on family harmony through self-efficacy and ego depletion. However, the research still just explores the linear relationship between proactive connectivity behaviors on the personal resources of employees and family outcomes. In the future, we can explore whether there is an inverted U-shaped relationship between proactive connectivity behaviors and work or family domain outcomes, that is, to study the different degrees of proactive connectivity behavior. Specifically, Individuals can keep energetic and bring high self-efficacy in the process of proactive work connectivity. However, in the long run, how excessive proactive work connectivity behaviors will affect employees' work and life needs to be further discussed. Second, although this study examined the moderating effect of family support, a family context resource, on the relationship between work connectivity behaviors and personal resources, there may be other moderating mechanisms. Future research can further improve the boundary conditions of the influencing mechanism of work connectivity behaviors from the perspective of personal traits, such as time management (Fenner and Renn, 2010), boundary segmentation preference (Andrade and Matias, 2021), etc. In addition, the research can also choose the variables of work in the selection of outcome variables, such as employee creativity. Third, although this study adopted a three-wave multi-time point questionnaire collection method to reduce the common method bias that may be caused by cross-sectional data, the samples were from a single source and were all self-reported by employees. Future research may consider multiple sources and allow family members to evaluate variables in the household domain to enrich the validity of sample measurement. In addition, this study used cross-sectional self-report data. Thus, the relationships in this study do not indicate causality. In future studies, experimental methods can be used to further verify the causal relationship, and the experience sampling method can also be considered to further improve the research design.

## Data availability statement

The original contributions presented in the study are included in the article/Supplementary material, further inquiries can be directed to the corresponding author.

## Ethics statement

The studies involving human participants were reviewed and approved by Zhongnan University of Economics and Law. The patients/participants provided their written informed consent to participate in this study. Written informed consent was obtained from the individual(s) for the publication of any potentially identifiable images or data included in this article.

## Author contributions

HH developed the theoretical model and wrote the manuscript. DL was responsible for the data collection as well as for the application of analytical tools. YZ and PZ participated in revising the manuscript. All authors contributed to the article and approved the submitted version.

## References

[B1] Andrade C. Matias M. (2021). Work-related ICT use during off-job time, technology to family conflict and segmentation preference: a study with two generations of employees. Inf. Commun. Soc. 25, 2162–2171. 10.1080/1369118X.2021.1933564

[B2] Bandura A. (1986). Social Foundations of Thought and Action: A Social Cognitive Theory. Englewood Cliffs, NJ: Prentice Hall.

[B3] Bardoel E. A. Pettit T. M. De Cieri H. McMillan L. (2014). Employee resilience: an emerging challenge for HRM. Asia Pac. J. Hum. Resour. 52, 279–297. 10.1111/1744-7941.12033

[B4] Boswell W. R. Olson-Buchanan J. B. (2007). The use of communication technologies after hours: the role of work attitudes and work-life conflict. J. Manage. 33, 592–610. 10.1177/0149206307302552

[B5] Carvalho V. S. Correia I. Chambel M. J. (2021). Is it ok to be connected outside the office? The impact on well-being at work and the mediating role of the work and family relationship. Int. J. Organ. Anal. 30, 1856–1856. 10.1108/IJOA-01-2021-2577

[B6] Chen Z. Ellis A. M. (2021). Crossover of daily job stressors among dual-career couples: A dyadic examination. J. Organ. Behav. 42, 668–683. 10.1002/job.2520

[B7] Crawford E. R. LePine J. A. Rich B. L. (2010). Linking job demands and resources to employee engagement and burnout: a theoretical extension and meta-analytic test. J. Appl. Psychol. 95, 834–848. 10.1037/a0019364 20836586

[B8] Daniel C. Gentina E. Mesmer-Magnus J. (2022). Mindfulness buffers the deleterious effects of workaholism for work-family conflict. Soc. Sci. Med. 306, 115118. 10.1016/j.socscimed.2022.115118 35696778

[B9] Derks D. Bakker A. B. Peters P. van Wingerden P. (2016). Work-related smartphone use, work–family conflict and family role performance: the role of segmentation preference. Hum. Relat. 69, 1045–1068. 10.1177/0018726715601890

[B10] Dumas T. L. Perry-Smith J. E. (2018). The paradox of family structure and plans after work: Why single childless employees may be the least absorbed at work. Acad. Manage. J. 61, 1231–1252. 10.5465/amj.2016.0086

[B11] Fenner G. H. Renn R. W. (2010). Technology-assisted supplemental work and work-to-family conflict: the role of instrumentality beliefs, organizational expectations and time management. Hum. Relat. 63, 63–82. 10.1177/0018726709351064

[B12] Fornell C. Larcker D. F. (1981). Evaluating structural equation models with unobservable variables and measurement error. J. Mark. Res. 18, 39–50. 10.1177/00222437810180010

[B13] Fujimoto Y. Ferdous A. S. Sekiguchi T. Sugianto L. F. (2016). The effect of mobile technology usage on work engagement and emotional exhaustion in Japan. J. Bus. Res. 69, 3315–3323. 10.1016/j.jbusres.2016.02.013

[B14] Greenhaus J. H. Powell G. N. (2006). When work and family are allies: a theory of work-family enrichment. Acad. Manage Rev. 31, 72–92. 10.5465/amr.2006.19379625

[B15] Hagger M. S. Wood C. Stiff C. Chatzisarantis N. L. D. (2010). Ego depletion and the strength model of self-control: a meta-analysis. Psychol. Bull. 136, 495–525. 10.1037/a0019486 20565167

[B16] Halbesleben J. Neveu J.-P. Paustian-Underdahl S. Westman M. (2014). Getting to the “COR”: understanding the role of resources in conservation of resources theory. J. Manage. 40, 1334–1364. 10.1177/0149206314527130

[B17] Hammer L. B. Johnson R. C. Crain T. L. Bodner T. Kossek E. E. Davis K. D.. (2016). Intervention effects on safety compliance and citizenship behaviors: evidence from the work, family, and health study. J. Appl. Psychol. 101, 190–208. 10.1037/apl0000047 26348479 PMC4564872

[B18] Huo W. Xu X. Li X. Xie J. Sun L. (2022). Work-related use of information and communication technologies after-hours (W_ICTs) and employee innovation behavior: a dual-path model. Inf. Technol. People. 10.1108/ITP-06-2021-0500

[B19] Ip P.-K. (2014). Harmony as happiness? social harmony in two chinese societies. Soc. Indic. Res. 117, 719–741. 10.1007/s11205-013-0395-7

[B20] Judge T. A. Bono J. E. (2001). Relationship of core self-evaluations traits—self-esteem, generalized self-efficacy, locus of control, and emotional stability—with job satisfaction and job performance: a meta-analysis. J. Appl. Psychol. 86, 80–92. 10.1037/0021-9010.86.1.80 11302235

[B21] Kang Y. J. Peng J. (2019). Benefits and costs of servant leadership behavior: a work-home resource model perspective. Acta Psychologica Sinica. 51, 227–237. 10.3724/SP.J.1041.2019.00227

[B22] Kavikondala S. Stewart S. M. Ni M. Y. Chan B. H. Y. Lee P. H. Li K.-K.. (2016). Structure and validity of family harmony scale: an instrument for measuring harmony. Psychol. Assess. 28, 307–318. 10.1037/pas0000131 26146946

[B23] Kenny D. A. Korchmaros J. D. Bolger N. (2003). Lower level mediation in multilevel models. Psychol. Methods. 8, 115–128. 10.1037/1082-989X.8.2.115 12924810

[B24] Khalid J. Weng Q. D. Luqman A. Rasheed M. I. Hina M. (2021). After-hours work-related technology use and individuals' deviance: the role of other-initiated vs. self-initiated interruptions. Inf. Technol. People. 35, 1955–1979. 10.1108/ITP-03-2020-0136

[B25] Lee E. S. Shin Y.-J. (2017). Social cognitive predictors of Korean secondary school teachers' job and life satisfaction. J. Vocat. Behav. 102, 139–150. 10.1016/j.jvb.2017.07.008

[B26] Lee S. Zhou Z. E. Xie J. Guo H. (2021). Work-related use of information and communication technologies after hours and employee fatigue: the exacerbating effect of affective commitment. J. Manag. Psychol. 36, 477–490. 10.1108/JMP-12-2019-0677

[B27] Lin S.-H. Johnson R. E. (2015). A suggestion to improve a day keeps your depletion away: Examining promotive and prohibitive voice behaviors within a regulatory focus and ego depletion framework. J. Appl. Psychol. 100, 1381–1397. 10.1037/apl0000018 25706447

[B28] Ma H. Y. Xie J. L. Tang H. Y. Shen C. G. Zhang X. X. (2016). Relationship between working through information and communication technologies after hours and well-being among Chinese dual-earner couples: a spillover-crossover perspective. Acta Psychologica Sinica. 48, 48–58. 10.3724/SP.J.1041.2016.00048

[B29] MacKinnon D. P. Krull J. L. Lockwood C. M. (2000). Equivalence of the mediation, confounding and suppression effect. Prevent. Sci. 1, 173–181. 10.1023/A:1026595011371 11523746 PMC2819361

[B30] Mazmanian M. Orlikowski W. J. Yates J. (2013). The autonomy paradox: the implications of mobile email devices for knowledge professionals. Organizat. Sci. 24, 1337–1357. 10.1287/orsc.1120.0806

[B31] McNatt D. B. Judge T. A. (2008). Self-efficacy intervention, job attitudes, and turnover: a field experiment with employees in role transition. Hum. Relat. 61, 783–810. 10.1177/0018726708092404

[B32] Ohly S. Latour A. (2014). Work-related smartphone use and well-being in the evening: the role of autonomous and controlled motivation. J. Pers. Psychol. 13, 174. 10.1027/1866-5888/a000114

[B33] Park Y. Fritz C. (2015). Spousal recovery support, recovery experiences, and life satisfaction crossover among dual-earner couples. J. Appl. Psychol. 100, 557–566. 10.1037/a0037894 25222524

[B34] Piazza C. F. (2007). Workplace Connectivity: A Hidden Ethical Dilemma. California: Business and Organizational Ethics Partnership Markkula Center for Applied Ethics of Santa Clara University.

[B35] Podsakoff P. M. MacKenzie S. B. Lee J.-Y. Podsakoff N. P. (2003). Common method biases in behavioral research: a critical review of the literature and recommended remedies. J. Appl. Psychol. 88, 879–903. 10.1037/0021-9010.88.5.879 14516251

[B36] Preacher K. J. Hayes A. F. (2008). Asymptotic and resampling strategies for assessing and comparing indirect effects in multiple mediator models. Behav. Res. Methods 40, 879–891. 10.3758/BRM.40.3.879 18697684

[B37] Ragsdale J. M. Hoover C. S. (2016). Cell phones during nonwork time: a source of job demands and resources. Comput. Human Behav. 57, 54–60. 10.1016/j.chb.2015.12.017

[B38] Reinke K. Ohly S. (2021). Double-edged effects of work-related technology use after hours on employee well-being and recovery: the role of appraisal and its determinants. Ger. J. Hum. Resour. Manag. 35, 224–248. 10.1177/2397002221995797

[B39] Ren S. Hu J. Tang G. Chadee D. (2021). Digital connectivity for work after hours: Its curvilinear relationship with employee job performance. Pers. Psychol. 10.1111/peps.12497

[B40] Richardson and Benbunan-Fich. (2011). Examining the antecedents of work connectivity behavior during non-work time. Inf. Organizat. 21, 142–160. 10.1016/j.infoandorg.2011.06.002

[B41] Richardson K. M. Thompson C. A. (2012). High tech tethers and work-family conflict: a conservation of resources approach. Eng. Manag. Res. 1, p29. 10.5539/emr.v1n1p29

[B42] Schaufeli W. B. Taris T. W. (2014). “A critical review of the job demands-resources model: Implications for improving work and health,” in Bridging occupational, organizational and public health: A transdisciplinary approach. Berlin, Germany: Springer Science + Business Media. p. 43–68. 10.1007/978-94-007-5640-3_4

[B43] Schlachter S. McDowall A. Cropley M. Inceoglu I. (2018). Voluntary work-related technology use during non-work time: a narrative synthesis of empirical research and research agenda. Int. J. Manag. Rev. 20, 825–846. 10.1111/ijmr.12165

[B44] Schulte-Braucks J. Baethge A. Dormann C. Vahle-Hinz T. (2019). Get even and feel good? Moderating effects of justice sensitivity and counterproductive work behavior on the relationship between illegitimate tasks and self-esteem. J. Occup. Health Psychol. 24, 241. 10.1037/ocp0000112 29683712

[B45] Schwarzer R. Bäßler J. Kwiatek P. Schröder K. Zhang J. X. (1997a). The assessment of optimistic self-beliefs: comparison of the German, Spanish, and Chinese versions of the general self-efficacy scale. Appl. Psychol. 46, 69–88. 10.1111/j.1464-0597.1997.tb01096.x

[B46] Schwarzer R. Born A. Iwawaki S. Lee Y.-M.. (1997b). The assessment of optimistic self-beliefs: comparison of the Chinese, Indonesian, Japanese, and Korean versions of the general self-efficacy scale. Psychologiat. 40, 1–13.

[B47] Seiger C. P. Wiese B. S. (2009). Social support from work and family domains as an antecedent or moderator of work–family conflicts? J. Vocat. Behav. 75, 26–37. 10.1016/j.jvb.2009.03.00124782044

[B48] Siu O. Lu J. Brough P. Lu C. Bakker A. B. Kalliath T.. (2010). Role resources and work–family enrichment: the role of work engagement. J. Vocat. Behav. 77, 470–480. 10.1016/j.jvb.2010.06.007

[B49] Sonnentag S. (2018). The recovery paradox: portraying the complex interplay between job stressors, lack of recovery, and poor well-being. Res. Organ. Behav. 38, 169–185. 10.1016/j.riob.2018.11.002

[B50] Tang S. Siu O. Cheung F. (2014). A study of work–family enrichment among Chinese employees: The mediating role between work support and job satisfaction. Appl. Psychol. 63, 130–150. 10.1111/j.1464-0597.2012.00519.x

[B51] Tangney J. P. Baumeister R. F. Boone A. L. (2004). High self-control predicts good adjustment, less pathology, better grades, and interpersonal success. J. Pers. 72, 271–322. 10.1111/j.0022-3506.2004.00263.x 15016066

[B52] ten Brummelhuis L. L. Bakker A. B. (2012). A resource perspective on the work–home interface: the work–home resources model. Am. Psycholog. 67, 545–556. 10.1037/a0027974 22506688

[B53] Ter Hoeven C. L. van Zoonen W. Fonner K. L. (2016). The practical paradox of technology: the influence of communication technology use on employee burnout and engagement. Commun. Monogr. 83, 239–263. 10.1080/03637751.2015.1133920 27226694 PMC4867864

[B54] Wan M. Shaffer M. A. Lau T. Cheung E. (2019). The knife cuts on both sides: examining the relationship between cross-domain communication and work–family interface. J. Occup. Organ. Psychol. 92, 978–1019. 10.1111/joop.12284

[B55] Watson D. Wiese D. Vaidya J. Tellegen A. (1999). The two general activation systems of affect: structural findings, evolutionary considerations, and psychobiological evidence. J. Pers. Soc. Psychol. 820–838. 10.1037/0022-3514.76.5.820

[B56] Wen Z. L. Ye B. J. (2014). Analyses of mediating effects: the development of methods and models. Adv. Cogn. Psychol. 22, 731–745. 10.3724/SP.J.1042.2014.00731

[B57] Xie J. Ma H. Zhou Z. E. Tang H. (2018). Work-related use of information and communication technologies after hours (W_ICTs) and emotional exhaustion: a mediated moderation model. Comput. Human Behav. 79, 94–104. 10.1016/j.chb.2017.10.023

[B58] Yang Y. Yan R. Meng Y. (2022). Can't disconnect even after-hours: how work connectivity behavior after-hours affects employees' thriving at work and family. Front. Psychol. 13, 865776. 10.3389/fpsyg.2022.865776 35356326 PMC8959651

