# Supplementary material for: The spillover effect of work connectivity behaviors on employees' family: Based on the perspective of work-home resource model
Source: Front Psychol. 2023 Feb 9;14:1067645. doi: 10.3389/fpsyg.2023.1067645 (PMC9947708; doi:10.3389/fpsyg.2023.1067645)
Supplement: Supplementary file 1 [file Table_1.docx]

**APPENDIX**

| **Constructs** | **Items** |
| --- | --- |
| proactive work connectivity behaviors | When I fall behind in my work during the day, I proactively work hard at home at night or on weekends to get caught up by using my cell phone or computer |
|  | when I return home from work, I proactively use my cell phone or computer for work-related tasks |
|  | I proactively perform job-related tasks at home at night or on weekends using my cell phone, or computer. |
|  | I feel my cell phone or computer is helpful in enabling me to work at home at night or on weekends |
|  | When there is an urgent issue or deadline at work, I tend to proactively bring work-related tasks home at night or on weekends and use my cell phone or computer to perform work-related tasks |
|  | When I am at home at night or on weekends, I proactively pay attention to work-related notifications through my cell phone or computer |
| Passive work connectivity behaviors | When I fall behind in my work during the day, my leader usually asks me to work hard at home at night or on weekends to get caught up by using my cell phone or computer |
|  | when I return home from work, I passively use my cell phone or computer for work-related tasks |
|  | My leader usually asks me to deal with work-related issues at home at night or on weekends |
|  | When I am at home at night or on weekends, I am disgusted that my colleagues or leaders contact me through cell phone or computer to deal with work-related matters |
|  | When there is an urgent issue or deadline at work, my leader usually asks me to bring work-related tasks home at night or on weekends and use my cell phone or computer to perform work-related tasks |
|  | When I am at home at night or on weekends, my leader asks me to pay attention to work-related notifications through my cell phone or computer |
| Self-efficacy | I can always manage to solve difficult problems if I try hard enough |
|  | If someone opposes me, I can find the means and ways to get what I want |
|  | It is easy for me to stick to my aims and accomplish my goals |
|  | I am confident that I could deal efficiently with unexpected events |
|  | Thanks to my resourcefulness, I know how to handle unforeseen situations |
|  | I can solve most problems if I invest the necessary effort |
|  | I can remain calm when facing difficulties because I can rely on my coping abilities |
|  | When I am confronted with a problem, I can usually find several solutions |
|  | If I am in trouble, I can usually think of a solution |
|  | I can usually handle whatever comes my way |
| ego depletion | I feel drained |
|  | My mind feels unfocused |
|  | It would take a lot of effort for me to concentrate on something |
|  | My mental energy is running low |
|  | I feel like my willpower is gone |
| family support | how much the family members provide you with encouragement |
|  | how much the family members provide you with useful information |
|  | how much the family members say things that raise your self-confidence |
|  | how much the family members listen to you when you need to talk |
|  | how much the family members show that they care about you as a person |
|  | how much the family members understand the way you think and feel about things |
|  | how much the family members talk with you when you are upset |
|  | how much the family members help you understand and sort things out |
|  | how much the family members provide you with direct help |
|  | how much the family members make you feel you can rely on them |
| family harmony | My family functions well for all members |
|  | My family’s day-to-day interactions are peaceful |
|  | My family members accommodate each other |
|  | I am proud of my family |
|  | My family is harmonious |
